# Partitioning the sources of demographic variation reveals density-dependent nest predation in an island bird population

**DOI:** 10.1002/ece3.1127

**Published:** 2014-06-06

**Authors:** Helen R Sofaer, T Scott Sillett, Kathryn M Langin, Scott A Morrison, Cameron K Ghalambor

**Affiliations:** 1 Graduate Degree Program in Ecology and Biology Department, Colorado State University1878 Campus Delivery, Fort Collins, Colorado, 80523; 2 Migratory Bird Center, Smithsonian Conservation Biology Institute, National Zoological ParkMRC 5503, Washington, District of Columbia, 20013-7012; 3 The Nature Conservancy201 Mission St, 4th Floor, San Francisco, California, 94105

**Keywords:** Demography, density dependence, fecundity, island syndrome, nest predation, zero-inflated model

## Abstract

Ecological factors often shape demography through multiple mechanisms, making it difficult to identify the sources of demographic variation. In particular, conspecific density can influence both the strength of competition and the predation rate, but density-dependent competition has received more attention, particularly among terrestrial vertebrates and in island populations. A better understanding of how both competition and predation contribute to density-dependent variation in fecundity can be gained by partitioning the effects of density on offspring number from its effects on reproductive failure, while also evaluating how biotic and abiotic factors jointly shape demography. We examined the effects of population density and precipitation on fecundity, nest survival, and adult survival in an insular population of orange-crowned warblers (*Oreothlypis celata*) that breeds at high densities and exhibits a suite of traits suggesting strong intraspecific competition. Breeding density had a negative influence on fecundity, but it acted by increasing the probability of reproductive failure through nest predation, rather than through competition, which was predicted to reduce the number of offspring produced by successful individuals. Our results demonstrate that density-dependent nest predation can underlie the relationship between population density and fecundity even in a high-density, insular population where intraspecific competition should be strong.

## Introduction

Quantifying the demographic effects of population density is essential for understanding how populations are regulated (Murdoch 1994), but poses a major research challenge because population density can act via multiple mechanisms and in concert with other ecological factors (Ostfeld and Canham 1995; Hixon and Carr 1997; Forchhammer et al. 1998). For example, population density can shape fecundity through increased resource competition (Rodenhouse et al. 2003) or increased density-dependent offspring mortality (Arcese et al. 1992). Density-dependent reductions in survival can act on both juveniles (Clutton-Brock et al. 1987; Gaillard et al. 1998; Harms et al. 2000) and adults (Forrester 1995; Frederiksen and Bregnballe 2000) through altered rates of starvation, predation, parasitism, or disease (Dempster 1971; Walde and Murdoch 1988; Hochachka and Dhondt 2000; Holbrook and Schmitt 2002). Other ecological factors, such as food abundance and weather, exhibit the same potential to affect demography via multiple mechanisms, and also mediate the effects of population density on vital rates (Coulson et al. 2001; Sillett et al. 2004). Therefore, developing a mechanistic understanding of demographic variation requires partitioning the multiple processes by which ecological factors shape patterns of survival and reproduction.

The mechanisms by which population density influences fecundity are often obscured in natural populations, in part because fecundity is comprised of several components. Fecundity is defined as the number of young produced over a breeding season, and variation in this demographic rate arises because (1) many individuals fail to reproduce successfully and (2) successful individuals vary in the number of young they produce (Clutton-Brock 1988; Newton 1998). However, we rarely know whether the ecological processes that cause reproductive failure, here defined as producing no young over a breeding season, are distinct from those affecting offspring number. For example, studies of birds show that while food availability is often the most important factor limiting the number of offspring produced by successful individuals (Lack 1947; Martin 1987; Godfray et al. 1991; Nagy and Holmes 2005; Sofaer et al. 2013), low food abundance can also lead to failure by causing individuals to forgo breeding (Southern 1970; Grant et al. 2000; Jenouvrier et al. 2003; Langin et al. 2009). Similarly, nest predation is the primary cause of avian nest and reproductive failure (Ricklefs 1969), but can also affect offspring number by influencing clutch size (Skutch 1949; Martin et al. 2000; Eggers et al. 2006) or the number of young fledged (Zanette et al. 2011). Because population density can alter food availability (Arcese and Smith 1988; Newton 1998) and nest predation risk (Gunnarsson and Elmberg 2008), it can act via either factor to affect offspring number or the probability of reproductive failure. Identifying and partitioning the processes that affect each component of fecundity can therefore reveal the mechanism through which density dependence shapes demographic variation.

Island populations have been used as model systems to investigate the mechanistic basis of demographic variation. These studies typically highlight the importance of both competition (Blondel 2000; Brouwer et al. 2009; Nevoux et al. 2011) and the abiotic factors affecting food abundance (Grant et al. 2000). Intraspecific competition is thought to be strong in island populations because they occur at high densities relative to their mainland counterparts and are often characterized as having fewer predators (MacArthur et al. 1972; Yeaton 1974; Adler and Levins 1994). However, despite the past emphasis on density-dependent competition, nest survival (the probability a nest fledges one or more young) in island bird populations can also be density dependent (Arcese et al. 1992), and nest predation rates on islands may be no lower than those on mainlands (Covas 2012). These patterns emphasize the need to determine how different mechanisms of density dependence and the abiotic drivers of food abundance jointly shape the demography of insular populations.

**Figure 1:**
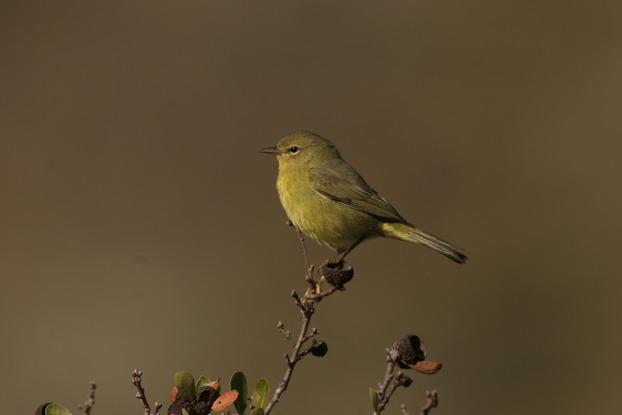
Orange-crowned warbler (*Oreothlypis celata*) on Santa Catalina Island, California. Photo by Dr. Moo-Boo Yoon.

Here, we analyze the ecological correlates of demographic variation in an island population of orange-crowned warblers (*Oreothlypis celata*; Fig. 1). Our study population breeds at a higher density than mainland populations (see below) and exhibits traits associated with increased intraspecific competition (e.g., high testosterone, Horton et al. 2010; high aggression, Yoon et al. 2012). We therefore expected density-dependent competition to underlie demographic variation in this system, along with variation in rainfall, which is a driver of food abundance (Morrison and Bolger 2002; Sofaer et al. 2013). We studied the effects of population density and rainfall on two major vital rates: fecundity and annual adult survival. Our analysis of fecundity separated the processes affecting the offspring number of successful individuals from those affecting the probability of reproductive failure, allowing us to assess the demographic effects of intraspecific competition and nest predation. By partitioning the effects of different processes on a single demographic rate, we were able to clarify the mechanisms of density dependence and challenge our assumptions about the relative importance of competition and predation.

## Methods

### Field methods

We studied breeding orange-crowned warblers in Bulrush Canyon (33°20′N, 118°26′W) on Santa Catalina Island, CA (hereafter, Catalina Island), each spring from 2003 to 2009. Our study population belongs to the *O. c. sordida* subspecies, which is endemic to the California Channel Islands and remnant patches of coastal chaparral habitat on the adjacent mainland. Most adults within a 7-ha study area were captured in mist-nets using conspecific playback and given a unique combination of colored plastic leg bands and a numbered US Geological Survey aluminum band. This study area represented the central core of our study plot, which was intensively surveyed in all years. Each March, surveys were conducted throughout the study area and adjoining habitat to resight color-banded individuals for analyses of survival. We observed breeding pairs every 1–3 days throughout each breeding season to map territory boundaries relative to a 25 × 25 m grid system, and to locate and monitor all nesting attempts, including renesting and double brooding (see Sofaer et al. 2013). The number of territorial pairs in our study area ranged from 23 (3.4 territories/ha) to 43 (6.3 territories/ha), and for each pair, we calculated the distance to the nearest neighbor (m) based on the center of each territory polygon, as defined in ArcMap (ESRI, Inc., Redlands, CA).

### Fecundity and nest survival

We quantified the annual fecundity of 181 territorial warbler pairs. In order to exclude pairs that may have had an undetected renesting attempt, we limited our fecundity analysis to pairs that either successfully fledged young at any time during the breeding season or had an active, monitored nest after April 15th; birds that failed after this date were unlikely to successfully renest. However, our analysis of fecundity did include closely monitored pairs without any active nests in 2007, when an extreme drought caused nearly all pairs to skip breeding and only one monitored pair laid eggs (Langin et al. 2009); no data from that year were included in our analysis of nest survival. The average number of pairs included in our fecundity analysis each year was 26 (range: 13–33 pairs; Table S1). We calculated annual fecundity of each pair as the total number of young fledged from all nesting attempts in a given year.

Before analyzing the fecundity data, we visually inspected spatial plots and variograms to evaluate the degree of spatial autocorrelation in fecundity across our study area. A bubble plot of fecundity was created using the ggplot2 package in R (Wickham 2009; R Development Core Team 2012), and showed no evidence of variation in the distribution of fecundity across space (Fig. S1). In addition, variograms produced by combining data from all years (Fig. S2), as well as those based on each year alone, showed little to no increase in the semivariance with increasing distance. Variograms were produced using the geoR package in R (Diggle and Ribeiro 2007). Because these plots showed only weak evidence of spatial autocorrelation in fecundity, we proceeded with analyses assuming independence between pairs in neighboring territories and between pairs breeding in similar locations in subsequent years.

A major goal of our study was to identify the processes via which population density and rainfall affected fecundity. To do so, we used zero-inflated models, which explicitly modeled fecundity as a mixture of two probability distributions: one describing the probability of failure and one describing the number of offspring produced. Zero-inflated models appropriately handle data with a surplus of zeros (Lambert 1992) and so are increasingly used to analyze patterns of distribution and abundance (Fletcher et al. 2005; Martin et al. 2005; Zuur et al. 2012). Yet despite the potential value of these models for demographic analyses of populations in which many individuals either do not have the opportunity to breed or experience reproductive failure, few studies have used these methods to separate the ecological factors that affect offspring number from those that affect reproductive failure (Quintero et al. 2007; Walker et al. 2009; Smith et al. 2010).

In our study population, 52% (95 of 181) of pairs fledged no young in a given breeding season, and we used zero-inflated Poisson regression to study the sources of this demographic variation. While zeros can arise from the count-side process, the zero-side process models effects that generate a higher proportion of zeros than would be expected from a given Poisson distribution. For example, a Poisson distribution with a mean of 2.9 (the mean number of young fledged by successful pairs; see below) should yield data with 5.5% zeros, whereas over half of our observations were zeros. The probability of a zero was modeled as a binomial distribution with a logit link, while the count was modeled as a Poisson distribution with a log link.

We evaluated how rainfall and breeding density (number of territorial pairs per ha) affected both the zero-side and count-side processes. We calculated breeding season precipitation as the total rainfall from November through April recorded at a weather station at Middle Ranch, Catalina Island (33°21′N, 118°26′W). This measure of precipitation was positively correlated with the primary food source for the warblers (insect larvae), clutch size, and breeding season length, and negatively correlated with first clutch initiation date (Sofaer et al. 2013). To account for the shared density and rainfall values for pairs in the same year, we initially fit a normally distributed random effect of year on both the zero and count sides of the model. However, the count-side random effect was estimated at zero in the full model, so we refit all models with a random effect only on the zero side. We considered the following four fixed-effect model structures: intercept only, a rainfall effect, a breeding density effect, and additive effects of rainfall and breeding density. We fit all combinations of these four model structures on both the count-side and zero-side processes, for a total of 16 possible models (Table 1), but did not have sufficient years of data to consider interactions between breeding density and rainfall. For model selection, we used Akaike's information criteria adjusted for small sample sizes (AIC_c_; (Burnham and Anderson 2002). Fecundity models were fit using the NLMIXED procedure in SAS (SAS Institute 2008). For all analyses, we report parameter estimates, standard errors, and confidence intervals based on the top model in which each covariate appeared.

**Table 1:** Model selection results for zero-inflated mixed models of fecundity indicated strong support for the effects of breeding density (bd) and precipitation (precip) on the probability of fledging zero young. We considered all possible additive model structures on both the count side and zero side, including intercept-only (.) fixed-effect structures. All models contained a normally distributed random effect of year on the zero side

Count-side model	Zero-side model	AIC_c_	ΔAIC_c_	Weight	−2log(L)	*k*
bd	bd + precip	464.02	0	0.33	451.53	6
.	bd + precip	464.50	0.48	0.26	454.16	5
bd + precip	bd + precip	464.58	0.56	0.25	449.93	7
precip	bd + precip	466.24	2.22	0.11	453.76	6
bd	bd	471.63	7.61	0.01	461.29	5
bd + precip	bd	471.65	7.63	0.01	459.17	6
.	bd	472.06	8.04	0.01	463.84	4
bd	precip	472.25	8.23	0.01	461.90	5
bd + precip	precip	472.98	8.96	0	460.50	6
.	precip	473.34	9.32	0	465.11	4
precip	bd	473.57	9.55	0	463.23	5
bd	.	474.84	10.82	0	466.61	4
bd + precip	.	475.07	11.05	0	464.72	5
precip	precip	475.15	11.13	0	464.81	5
.	.	475.54	11.52	0	469.41	3
precip	.	477.16	13.14	0	468.93	4

Our zero-inflated models indicated that breeding density affected the probability of fledging no young (see Results); therefore, to distinguish the effects of population density on breeding and/or renesting opportunities from its effects on nest predation, we examined how breeding density, precipitation, and other factors were correlated with daily nest survival probability. We limited this analysis to nests that were either successful or depredated because we were interested in factors affecting nest predation, rather than other sources of nest failure (i.e., abandonment, weather, and starvation), and because nest predation accounted for 80% of failures once at least one egg had been laid. We checked nest contents and observed adult behavior to assign a nest fate to all nests included in our analysis. For each breeding pair in each year, we included the earliest successful or depredated nest in which at least one egg was laid (*n* = 142 nests; mean per year = 24 nests); this restriction was imposed because nests from the same pair in the same year would not be independent. Following Rotella et al. (2004, 2007), we modeled daily nest survival using a generalized mixed model assuming a binomial distribution. Each day a nest was known to be active and each interval over which a nest failed contributed one survival interval to our analysis (*n* = 1983 intervals). All models were fit with a logit link function and included a normally distributed random effect of year because the nests from each year shared the same breeding density and level of precipitation. We built models including all additive combinations of the following five fixed effects: precipitation, date, nest height (Peluc et al. 2008), breeding density, and nearest neighbor distance (Table S2). The number of pairs per ha represented plot-level density effects, whereas nearest neighbor distance was used to assess the effects of local density (these two measures of density were not strongly correlated; *r* = −0.28). We used AIC_c_ for model selection. All nest survival models were fit using the NLMIXED procedure in SAS (SAS Institute 2008). Standard errors and confidence intervals were estimated with the delta method (Powell 2007; Cooch and White 2012).

### Adult survival

We fit Cormack–Jolly–Seber models (Lebreton et al. 1992) to estimate annual apparent survival (*ϕ*) and recapture (*p*) probabilities of territorial adults (*n* = 197) from March to the following March. We evaluated how apparent survival was influenced by conditions across the annual cycle by considering models with effects from both the breeding and nonbreeding seasons. Warblers breeding on Catalina Island largely winter on the adjacent mainland, so our models included covariates describing mainland conditions during the nonbreeding season. We considered all possible additive combinations of the following factors (described below) on annual apparent survival probability (*ϕ*): sex, breeding density, November–April precipitation, winter population density, and winter precipitation on the mainland (Table S3). All models included effects of sex and year on the resighting probability, *p*, and were fit using a logit link function in Program MARK (White and Burnham 1999) and ranked based on AIC_c_.

We calculated an index of winter warbler density based on data from the Christmas Bird Count (hereafter CBC; National Audubon Society 2010), a 1-day count that occurs between mid-December and early January. Volunteers follow designated routes within 24-km radius count circles and record all birds observed that day; the location of each count circle is consistent between years. Groups of participants, known as parties, search for birds, and effort is measured as the total number of party hours (Dunn et al. 2005). We summarized data from count circles in the regular wintering range of *O. c. sordida*, which includes coastal habitats in northern Baja California and five southern California counties: Los Angeles, Orange, San Diego, Santa Barbara, and Ventura (K. Garrett, pers. comm.). We included count circles where at least one orange-crowned warbler was detected in all years between the winters of 2003–04 and 2009–10 (*n* = 19 count circles). CBC data did not identify *O. celata* to subspecies, and counts likely included subspecies other than *O. c. sordida* (Dunn and Garrett 1997). Morphological differences between subspecies are relatively slight (Gilbert et al. 2010), so we assumed that *O. celata* subspecies were ecologically equivalent during the nonbreeding season and averaged the number of orange-crowned warblers seen per party hour across all counts to generate an index of winter density in each year (range 0.32–0.53 mean count per party hour). The use of CBC data necessarily entails several simplifying assumptions (reviewed in Dunn et al. 2005), and our use of the number of birds per party hour as an index of density assumed a linear relationship between effort and the resulting count (Link and Sauer 1999). This latter assumption appeared justified because the count data used in our analysis showed no evidence of reaching an asymptote with increasing effort.

We included precipitation in the survival models because our previous work in southern California demonstrated a strong correlation between rainfall and food abundance for songbirds (Morrison and Bolger 2002; Sofaer et al. 2013). We used yearly November to April precipitation totals for Catalina Island, described above, to represent conditions during the breeding season. Winter precipitation was calculated by averaging the total November to February rainfall at Western Regional Climate Center (http://www.wrcc.dri.edu) weather stations (*n* = 14) that had complete data and were located in the same five California counties as the CBC data. Wintering densities on the mainland were correlated with mainland precipitation during the survival interval (*r* = −0.75), so we did not build models that included both of these covariates.

## Results

### Breeding density and precipitation

To complement our demographic analyses, we examined the relationship between breeding density and precipitation to assess how breeding density responded to conditions in previous years. Breeding density was not strongly correlated with total November to April precipitation either in the same year (*r* = −0.13; Fig. S3A) or in the previous year (*r* = 0.26; Fig. S3B). In addition, our exploratory analyses were suggestive of population regulation, as breeding density in a given year was negatively correlated with density in the previous year (*r* = −0.79; Fig. S4A), perhaps reflecting the positive relationship between density and mean fecundity in the previous year (*r* = 0.76; Fig. S4B).

### Fecundity and nest survival

**Figure 2:**
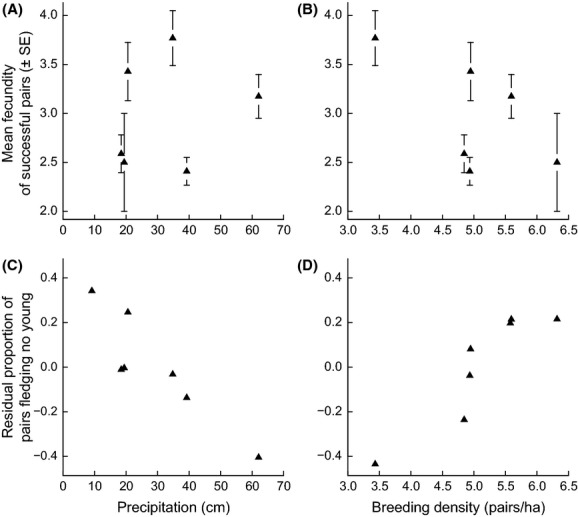
The fecundity of pairs that successfully fledged young was (A) not affected by precipitation and (B) weakly affected by breeding density. The proportion of pairs fledging no young in each year was strongly affected by both (C) precipitation and (D) breeding density. In (C), the *y*-axis coordinates are the residuals from a simple linear regression of the effects of density on the proportion of unsuccessful pairs, while in (D), they are the residuals from a simple linear regression of the effects of rainfall on the proportion of unsuccessful pairs. See text for formal zero-inflated analysis of fecundity.

Mean annual fecundity (±1 SE) was 1.40 ± 0.12 young fledged per pair per breeding season (*n* = 181 pairs). Excluding 2007, when no birds bred successfully, resulted in a mean annual fecundity estimate of 1.55 ± 0.13 young per pair (*n* = 163 pairs); the proportion of pairs fledging no young in each of these years ranged from 0.00 to 0.87 (Table S1). Birds that successfully fledged at least one offspring had a mean annual fecundity of 2.94 ± 0.11 young per pair (*n* = 86 pairs). Zero-inflated models found weak effects of rainfall and density on the number of offspring fledged (Fig. 2A and B), but strong effects of these factors on the probability of fledging no offspring (Fig. 2C and D). Birds were more likely to be unsuccessful in drier years (Fig. 2C; *β* = −0.07 ± 0.02; 95% CI: −0.11, −0.03) and in years with higher breeding density (Fig. 2D; *β* = 1.64 ± 0.45; 95% CI: 0.53, 2.75). Removing precipitation and breeding density from the top model increased the AIC_c_ value by 7.61 and 8.23, respectively, greatly reducing model fit (Table 1). We found less support for effects of rainfall and breeding density on the count side of the model, that is, on the number of offspring fledged (Table 1). The top model contained a breeding density effect on the count side, but a model without this effect received equivalent support (ΔAIC_c_ = 0.48; Table 1). Furthermore, the confidence interval for the count-side breeding density parameter (±1 SE) included zero (*β* = −0.14 ± 0.09; 95% CI: −0.36, 0.07), and support for this effect appeared to be influenced by 1 year of data (Fig. 2B). Similarly, a model including an effect of precipitation on the count side also received equivalent support (ΔAIC_c_ = 0.56; Table 1), and here too the confidence interval for this parameter included zero (*β* = 0.006 ± 0.005; 95% CI: −0.006, 0.018). We therefore concluded that while support for effects of breeding density and rainfall on the probability of reproductive failure was strong, we had only weak evidence suggesting these variables influenced the number of young fledged from successful nests during our study.

**Figure 3:**
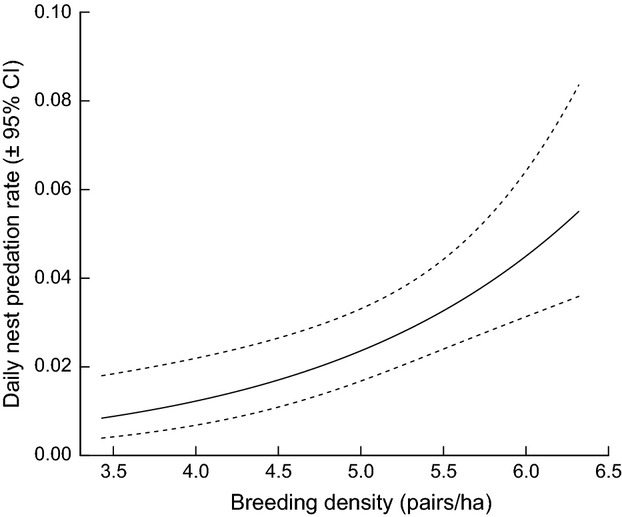
Daily nest predation rate increased with breeding density. Between 2003 and 2009, breeding density varied from 3.4 to 6.3 territories/ha.

Nest survival showed a strong pattern of density dependence. Daily survival probability was lower in years with a higher breeding density (Fig. 3, plotted as daily nest predation rate = 1 − daily nest survival rate; *β* = −0.67 ± 0.18; 95% CI: −1.13, −0.20); removing breeding density from the top model increased AIC_c_ by 7.45 (Table S2). Model selection indicated less support for the effects of nearest neighbor distance on nest survival (Table S2; *β* = −0.02 ± 0.02; 95% CI: −0.06, 0.02). We found little support for an effect of rainfall on nest survival probability (Table S2; *β* = −0.01 ± 0.01; 95% CI: −0.03, 0.01). Nest survival also declined as the season progressed (*β* = −0.03 ± 0.01; 95% CI: −0.05, −0.01) and increased with nest height, although zero was included near the boundary of the 95% confidence interval (*β* = 0.20 ± 0.11; 95% CI: −0.09, 0.50). Daily nest survival probability (±1 SE) was 0.974 ± 0.004 based on our top model and mean breeding density, date, and nest height values. To illustrate the magnitude of the effect of breeding density on the probability of nest predation, we raised the estimated daily nest survival rate to the 12th power (the length of the incubation period), and predicted 90.3% versus 50.7% nest survival over that period at the lowest versus the highest observed breeding densities.

### Adult survival

We found little evidence that precipitation or population density during either the breeding or wintering seasons affected annual adult survival probability, as the model with only a sex effect had equivalent statistical support as models containing rainfall and density effects (Table S3). Our confidence intervals included zero for the effects of breeding density (*β* = −0.53 ± 0.44; 95% CI: −1.39, 0.34), wintering density (*β* = −1.79 ± 1.68; 95% CI: −5.07, 1.50), November–April precipitation prior to the survival interval (precip *x*−1; *β* = 0.01 ± 0.01; 95% CI: −0.00, 0.03), and November–February precipitation during the survival interval (precip *x*; *β* = 0.01 ± 0.01; 95% CI: −0.01, 0.03). Estimated annual survival probability was higher for males (0.68 ± 0.03; 95% CI: 0.62, 0.73) than for females (0.56 ± 0.05; 95% CI: 0.47, 0.66).

## Discussion

We expected that intraspecific competition for food would underlie demographic variation in our study population and would reduce offspring number or lower adult survival. Like many insular animal populations (Adler and Levins 1994; Blondel 2000), orange-crowned warblers on Catalina Island breed at high densities (up to 6.3 pairs/ha versus up to 1.5 pairs/ha in mainland habitats; Gilbert et al. 2010), and male warblers exhibit increased levels of testosterone and aggression (Horton et al. 2010; Yoon et al. 2012). Nevertheless, breeding density had only a weak effect on the number of young produced by successful individuals (Fig. 2B) and was not correlated with annual adult survival (Table S3). Instead, breeding density affected the probability of reproductive failure (Fig. 2D) via density-dependent nest predation (Fig. 3). By partitioning the factors affecting reproductive failure from those affecting the fecundity of successful individuals, we were able to improve our mechanistic understanding of demographic variation and separate the effects of density-dependent nest predation, intraspecific competition, and precipitation.

Because we found that both breeding density and rainfall affected the probability of fledging zero young (Fig. 2C and D), we evaluated the influence of these factors on three major processes that determine whether a territorial individual will reproduce successfully in a given year. First, a bird must breed, rather than skip breeding for a year. Second, nest survival depends on attributes of the parent, nest site, and predator community (reviewed in Cresswell 1997). Third, following nest failure, birds either renest or suspend breeding and enter the nonbreeding period, a decision that repeats with each failed reproductive attempt. The probability of renesting depends on the timing of failure and may be affected by food availability or the energetic reserves of the breeding adult (Nagy and Holmes 2005; Arnold et al. 2010; Caldwell et al. 2013). Below, we discuss how the effects of rainfall and breeding density may have been mediated via these three sources of reproductive failure in *O. celata* on Catalina Island.

Rainfall likely influenced the probability of reproductive failure by altering birds' propensity to initiate breeding and to renest after nest failure. Indeed, the importance of rainfall was observed during a severe drought, which led the majority of territorial pairs to skip breeding in 2007; not a single pair successfully fledged young in that year (Fig. 2C at 9 cm of rainfall; Langin et al. 2009). Increased rainfall was also associated with longer breeding seasons (Sofaer et al. 2013), suggesting that the relationship between rainfall and food abundance led birds to increase their probability of renesting as precipitation increased. Rainfall was not correlated with daily nest survival probability (Table S2). We expected that rainfall would have had a stronger effect on the number of young fledged (Fig. 2A) because birds in this population lay larger clutches in wetter years (Sofaer et al. 2013). However, the relatively small magnitude of change in mean clutch size between years (range: 2.8–3.5 eggs, including all nests throughout the breeding season) could explain our failure to find the expected effects on offspring number.

Nest predation appeared to be the primary mechanism underlying the density-dependent increase in reproductive failure (Figs. 2D, 3). Breeding density had no apparent effects on the probability of breeding, as all territorial pairs attempted to breed in the year with the highest observed conspecific density. Breeding density also did not influence breeding season length; a post hoc analysis of breeding season length found that the addition of breeding density to a simple linear model including precipitation increased the AIC value by 1.74, and the confidence interval on the density effect included zero.

Density-dependent nest predation could arise from either functional or numeric responses of nest predators to increasing prey density (Holling 1959) or from a form of site dependence in which areas with lower nest predation risk are preferentially occupied (Rodenhouse et al. 2003; Emmering and Schmidt 2011). This latter mechanism could arise if warblers can accurately assess spatial variation in nest predation risk, but testing this hypothesis is difficult because the probability of nest predation risk may shift spatially between years so particular territories may not consistently be favored. An alternative explanation of our results, that the relationship between warbler density and nest predation rates simply reflected correlated numerical responses of warbler and nest predator populations to high productivity in wet years, is unlikely because warbler densities were not correlated with precipitation in the previous year (Fig. S3B). Nevertheless, tracking nest predator abundance would allow for an analysis of the relationship between predator density and nest predation rates, which remains understudied (Abrams and Ginzburg 2000; Oro et al. 2006; Schmidt et al. 2008).

There is a need to understand how reptilian and mammalian predators locate nests and allocate foraging effort (Weatherhead and Bloun-Demers 2004), particularly given the possibility that one or more of the island's predators may not simply find nests incidentally. Documented or likely nest predators in this system include gopher snakes (*Pituophis catenifer*), California kingsnakes (*Lampropeltis californiae*), island foxes (*Urocyon littoralis*), feral cats (*Felis catus*), deer mice (*Peromyscus maniculatus*), and black and Norwegian rats *Rattus rattus*, *R. norvegicus*; Catalina Island lacks avian nest predators such as jays (Peluc et al. 2008). Currently, there is no consensus about how differences in the type, diversity, and abundance of predator and prey species influence the likelihood of nest predation being density dependent, which has been found in several systems (Andersson and Wiklund 1978; Martin 1996; Lariviere and Messier 1998; Schmidt and Whelan 1999; Gunnarsson and Elmberg 2008) but not in others (Zimmerman 1984; O'Reilly and Hannon 1989; Reitsma 1992; Ackerman et al. 2004; Sillett and Holmes 2005). However, our study is one of the few that has both documented density-dependent nest predation in a natural population and also assessed whether nest predation underlies variation in fecundity, giving it the potential to regulate the focal population (Arcese et al. 1992; Tapper et al. 1996).

Density-dependent nest predation also has implications for the evolution and expression of nest-site selection. When a nest predator's functional response includes specializing on a set of nest-site characteristics, individuals may benefit by nesting in less-used sites, a process that can affect community composition and the evolution of nest-site selection (Martin 1988, 1996), although not all studies have found support for predator specialization (Reitsma and Whelan 2000; Rangen et al. 2001). Orange-crowned warblers on Catalina Island (*O. c. sordida*) exhibit an unusual amount of variation in nest height and nest-site location relative to most other *Oreothlypis*, including mainland populations of *O. celata*, which almost always nest on the ground. Warbler nest sites on Catalina Island range from ground nests that experience relatively high nest predation, to safer off-ground nests in shrubs and tree crowns (Peluc et al. 2008). Our results therefore raise the possibility that density-dependent nest predation could favor the diversification of nesting sites within a population, rather than solely between species. Additional research is needed to evaluate nest predators' search methods and functional responses, whether high warbler breeding densities on Catalina Island could have favored specialization by predators, and whether warblers gain any selective advantage from choosing atypical nest-site locations; support for this latter hypothesis would imply that nest predation is frequency dependent.

## Conclusions

Density-dependent nest predation appeared to be an important regulatory mechanism for *O. celata* on Catalina Island (Fig. 3), even though our study population bred at high densities and exhibited traits associated with strong intraspecific competition (Horton et al. 2010; Yoon et al. 2012). However, our research focused only on territory holders, and intraspecific competition for food and territories is likely to influence postfledging survival and recruitment in this population. Our ability to interpret patterns of density-dependent fecundity was facilitated by the use of zero-inflated models, which allowed us to separate the ecological factors affecting the count-side process (i.e., the number of young fledged) from those affecting the zero-side process (i.e., the probability of fledging no young). We suggest that the broader use of zero-inflated models will improve ecological inference about populations in which many individuals fail to successfully reproduce.

## Data Accessibility

Data are available from the Dryad Digital Repository doi:10.5061/dryad.m4fs7.

## Supporting Information

**Table S1.** Summary statistics describing mean annual variation in fecundity (total offspring fledged per pair). **Table S2.** Our generalized mixed models showed strong support for density-dependent effects on daily nest survival; at higher breeding densities daily nest survival was lower. **Table S3**. Model selection results for the top ten Cormack-Jolly-Seber mark-recapture models. **Figure S1**. A bubble plot of the annual fecundity of warbler pairs in our study area showed no consistent pattern in the distribution of fecundity across space. **Figure S2.** A variogram of fecundity showing weak evidence of spatial autocorrelation within our study area. **Figure S3**. Breeding density in a given year was not correlated with total November–April precipitation (A) in the same year or (B) in the previous year. **Figure S4**. Breeding density was (A) negatively correlated with breeding density in the previous year, and (B) positively correlated with mean fecundity in the previous year.

## References

[b1] Abrams PA, Ginzburg LR (2000). The nature of predation: prey dependent, ratio dependent or neither?. Trends Ecol. Evol.

[b2] Ackerman JT, Blackmer AL, Eadie JM (2004). Is predation on waterfowl nests density dependent? Tests at three spatial scales. Oikos.

[b3] Adler GH, Levins R (1994). The island syndrome in rodent populations. Q. Rev. Biol.

[b4] Andersson M, Wiklund CG (1978). Clumping versus spacing out - experiments on nest predation in fieldfares (*Turdus pilaris*. Anim. Behav.

[b5] Arcese P, Smith JNM (1988). Effects of population density and supplemental food on reproduction in song sparrows. J. Anim. Ecol.

[b6] Arcese P, Smith JNM, Hochachka WM, Rogers CM, Ludwig D (1992). Stability, regulation, and the determination of abundance in an insular song sparrow population. Ecology.

[b7] Arnold TW, Devries JH, Howerter DW (2010). Factors that affect renesting in mallards (*Anas platyrhynchos*. Auk.

[b8] Blondel J (2000). Evolution and ecology of birds on islands: trends and prospects. Vie Et Milieu-Life Environ.

[b9] Brouwer L, Tinbergen JM, Both C, Bristol R, Richardson DS, Komdeur J (2009). Experimental evidence for density-dependent reproduction in a cooperatively breeding passerine. Ecology.

[b10] Burnham KP, Anderson DR (2002). Model selection and multimodel inference: a practical information-theoretic approach.

[b11] Caldwell L, Bakker VJ, Sillett TS, Desrosiers MA, Morrison SA, Angeloni LM (2013). The influence of habitat and nest observation on the reproductive success of the island scrub-jay. Condor.

[b12] Clutton-Brock TH (1988). Reproductive success.

[b13] Clutton-Brock TH, Major M, Albon SD, Guinness FE (1987). Early development and population dynamics in red deer. I. Density-dependent effects on juvenile survival. J. Anim. Ecol.

[b14] Cooch EG, White GC (2012). http://www.phidot.org/software/mark/docs/book/.

[b15] Coulson T, Catchpole EA, Albon SD, Morgan BJT, Pemberton JM, Clutton-Brock TH (2001). Age, sex, density, winter weather, and population crashes in Soay sheep. Science.

[b16] Covas R (2012). Evolution of reproductive life histories in island birds worldwide. Proc. Biol. Sci.

[b17] Cresswell W (1997). Nest predation: the relative effects of nest characteristics, clutch size and parental behaviour. Anim. Behav.

[b18] Dempster JP (1971). Population ecology of cinnabar moth, *Tyria jacobaeae* L. (Lepidoptera, Arctiidae). Oecologia.

[b19] Development Core Team R (2012). R: A language and environment for statistical computing.

[b20] Diggle PJ, Ribeiro PJ (2007). Model-based geostatistics.

[b21] Dunn J, Garrett K (1997). A field guide to warblers of North America.

[b22] Dunn EH, Francis CM, Blancher PJ, Drennan SR, Howe MA, Lepage D (2005). Enhancing the scientific value of The Christmas Bird Count. Auk.

[b23] Eggers S, Griesser M, Nystrand M, Ekman J (2006). Predation risk induces changes in nest-site selection and clutch size in the Siberian jay. Proc. Biol. Sci.

[b91] Emmering QC, Schmidt KA (2011). Nesting songbirds assess spatial heterogeneity of predatory chipmunks by eavesdropping on their vocalizations. J. Anim. Ecol.

[b24] Fletcher D, MacKenzie D, Villouta E (2005). Modelling skewed data with many zeros: a simple approach combining ordinary and logistic regression. Environ. Ecol. Stat.

[b25] Forchhammer MC, Stenseth NC, Post E, Langvatn R (1998). Population dynamics of Norwegian red deer: density-dependence and climatic variation. Proc. Biol. Sci.

[b26] Forrester GE (1995). Strong density-dependent survival and recruitment regulate the abundance of a coral reef fish. Oecologia.

[b27] Frederiksen M, Bregnballe T (2000). Evidence for density-dependent survival in adult cormorants from a combined analysis of recoveries and resightings. J. Anim. Ecol.

[b28] Gaillard JM, Festa-Bianchet M, Yoccoz NG (1998). Population dynamics of large herbivores: variable recruitment with constant adult survival. Trends Ecol. Evol.

[b29] Gilbert WM, Sogge MK, Van Riper C, Poole A (2010). Orange-crowned Warbler (*Oreothlypis celata*. The birds of North America online.

[b30] Godfray HCJ, Partridge L, Harvey PH (1991). Clutch size. Annu. Rev. Ecol. Syst.

[b31] Grant PR, Grant BR, Keller LF, Petren K (2000). Effects of El Nino events on Darwin's finch productivity. Ecology.

[b32] Gunnarsson G, Elmberg J (2008). Density-dependent nest predation - an experiment with simulated Mallard nests in contrasting landscapes. Ibis.

[b33] Harms KE, Wright SJ, Calderon O, Hernandez A, Herre EA (2000). Pervasive density-dependent recruitment enhances seedling diversity in a tropical forest. Nature.

[b34] Hixon MA, Carr MH (1997). Synergistic predation, density dependence, and population regulation in marine fish. Science.

[b35] Hochachka WM, Dhondt AA (2000). Density-dependent decline of host abundance resulting from a new infectious disease. Proc. Natl Acad. Sci. USA.

[b36] Holbrook SJ, Schmitt RJ (2002). Competition for shelter space causes density-dependent predation mortality in damselfishes. Ecology.

[b37] Holling C (1959). The components of predation as revealed by a study of small-mammal predation of the European pine sawfly. Can. Entomol.

[b38] Horton BM, Yoon J, Ghalambor CK, Moore IT, Sillett TS (2010). Seasonal and population variation in male testosterone levels in breeding orange-crowned warblers (*Vermivora celata*. Gen. Comp. Endocrinol.

[b39] Jenouvrier S, Barbraud C, Weimerskirch H (2003). Effects of climate variability on the temporal population dynamics of southern fulmars. J. Anim. Ecol.

[b40] Lack D (1947). The significance of clutch size. Ibis.

[b41] Lambert D (1992). Zero-inflated Poisson regression, with an application to defects in manufacturing. Technometrics.

[b42] Langin KM, Sillett TS, Yoon J, Sofaer HR, Ghalambor CK, Damiani CC, Garcelon DK (2009). Reproductive consequences of an extreme drought for songbirds on Santa Catalina and Santa Cruz Islands. Proceedings of the seventh California Islands symposium.

[b43] Lariviere S, Messier F (1998). Effect of density and nearest neighbours on simulated waterfowl nests: can predators recognize high-density nesting patches?. Oikos.

[b44] Lebreton JD, Burnham KP, Clobert J, Anderson DR (1992). Modeling survival and testing biological hypotheses using marked animals - a unified approach with case-studies. Ecol. Monogr.

[b45] Link WA, Sauer JR (1999). Controlling for varying effort in count surveys - An analysis of Christmas Bird Count data. J. Agric. Biol. Environ. Stat.

[b46] MacArthur RH, Diamond JM, Karr JR (1972). Density compensation in island faunas. Ecology.

[b47] Martin TE (1987). Food as a limit on breeding birds - a life-history perspective. Annu. Rev. Ecol. Syst.

[b48] Martin TE (1988). On the advantage of being different - nest predation and the coexistence of bird species. Proc. Natl Acad. Sci. USA.

[b49] Martin TE (1996). Fitness costs of resource overlap among coexisting bird species. Nature.

[b50] Martin TE, Martin PR, Olson CR, Heidinger BJ, Fontaine JJ (2000). Parental care and clutch sizes in North and South American birds. Science.

[b51] Martin TG, Wintle BA, Rhodes JR, Kuhnert PM, Field SA, Low-Choy SJ (2005). Zero tolerance ecology: improving ecological inference by modelling the source of zero observations. Ecol. Lett.

[b52] Morrison SA, Bolger DT (2002). Variation in a sparrow's reproductive success with rainfall: food and predator-mediated processes. Oecologia.

[b53] Murdoch WW (1994). Population regulation in theory and practice. Ecology.

[b54] Nagy LR, Holmes RT (2005). Food limits annual fecundity of a migratory songbird: an experimental study. Ecology.

[b55] National Audubon Society (2010). http://www.christmasbirdcount.org.

[b56] Nevoux M, Gimenez O, Arlt D, Nicoll M, Jones C, Norris K (2011). Population regulation of territorial species: both site dependence and interference mechanisms matter. Proc. Biol. Sci.

[b57] Newton I (1998). Population limitation in birds.

[b58] O'Reilly P, Hannon SJ (1989). Predation of simulated willow ptarmigan nests - the influence of density and cover on spatial and temporal patterns of predation. Can. J. Zool.

[b59] Oro D, Martinez-Abrain A, Paracuellos M, Nevado JC, Genovart M (2006). Influence of density dependence on predator-prey seabird interactions at large spatio-temporal scales. Proc. Biol. Sci.

[b60] Ostfeld RS, Canham CD (1995). Density-dependent processes in meadow voles - an experimental approach. Ecology.

[b61] Peluc SI, Sillett TS, Rotenberry JT, Ghalambor CK (2008). Adaptive phenotypic plasticity in an island songbird exposed to a novel predation risk. Behav. Ecol.

[b62] Powell LA (2007). Approximating variance of demographic parameters using the delta method: a reference for avian biologists. Condor.

[b63] Quintero HE, Abebe A, Davis DA (2007). Zero-inflated discrete statistical models for fecundity data analysis in channel catfish, *Ictalurus punctatus*. J. World Aquaculture Soc.

[b64] Rangen SA, Clark RG, Hobson KA (2001). Predator responses to similarity and dispersion of artificial nest sites: implications for the structure of Boreal forest songbird communities. Auk.

[b65] Reitsma L (1992). Is nest predation density dependent - a test using artificial nests. Can. J. Zool.

[b66] Reitsma LR, Whelan CJ (2000). Does vertical partitioning of nest sites decrease nest predation?. Auk.

[b67] Ricklefs RE (1969). An analysis of nesting mortality in birds. Smithson. Contrib. Zool.

[b68] Rodenhouse NL, Sillett TS, Doran PJ, Holmes RT (2003). Multiple density-dependence mechanisms regulate a migratory bird population during the breeding season. Proc. Biol. Sci.

[b69] Rotella J, Dinsmore S, Shaffer T (2004). Modeling nest-survival data: a comparison of recently developed methods that can be implemented in MARK and SAS. Anim. Biodivers. Conserv.

[b70] Rotella J, Taper M, Stephens S, Lindberg M (2007). Extending methods for modeling heterogeneity in nest-survival data using generalized mixed models. Stud. Avian Biol.

[b71] SAS Institute (2008). SAS version 9.2.

[b72] Schmidt KA, Whelan CJ (1999). Nest predation on woodland songbirds: when is nest predation density dependent?. Oikos.

[b73] Schmidt KA, Rush SA, Ostfeld RS (2008). Wood thrush nest success and post-fledging survival across a temporal pulse of small mammal abundance in an oak forest. J. Anim. Ecol.

[b74] Sillett TS, Holmes RT, Greenberg RS, Marra PP (2005). Long-term demographic trends, limiting factors, and the strength of density dependence in a breeding population of a migratory songbird. Birds of two worlds: the ecology and evolution of temperate-tropical migration.

[b75] Sillett TS, Rodenhouse NL, Holmes RT (2004). Experimentally reducing neighbor density affects reproduction and behavior of a migratory songbird. Ecology.

[b76] Skutch AF (1949). Do tropical birds rear as many young as they can nourish. Ibis.

[b77] Smith S, Mang T, De Bellocq JG, Schaschl H, Zeitlhofer C, Hacklander K (2010). Homozygosity at a class II MHC locus depresses female reproductive ability in European brown hares. Mol. Ecol.

[b78] Sofaer HR, Sillett TS, Peluc SI, Morrison SA, Ghalambor CK (2013). Differential effects of food availability and nest predation risk on avian reproductive strategies. Behav. Ecol.

[b79] Southern HN (1970). The natural control of a population of Tawny owls (*Strix aluco*. J. Zool.

[b80] Tapper SC, Potts GR, Brockless MH (1996). The effect of an experimental reduction in predation pressure on the breeding success and population density of grey partridges Perdix perdix. J. Appl. Ecol.

[b81] Walde SJ, Murdoch WW (1988). Spatial density dependence in parasitoids. Annu. Rev. Entomol.

[b82] Walker M, Hall A, Anderson RM, Basanez MG (2009). Density-dependent effects on the weight of female *Ascaris lumbricoides* infections of humans and its impact on patterns of egg production. Parasit. Vectors.

[b83] Weatherhead PJ, Bloun-Demers G (2004). Understanding avian nest predation: why ornithologists should study snakes. J. Avian Biol.

[b84] White GC, Burnham KP (1999). Program MARK: survival estimation from populations of marked animals. Bird Stud.

[b85] Wickham H (2009). ggplot2: elegant graphics for data analysis.

[b86] Yeaton RI (1974). Ecological analysis of chaparral and pine forest bird communities on Santa Cruz Island and mainland California. Ecology.

[b87] Yoon J, Sillett TS, Morrison SA, Ghalambor CK (2012). Breeding density, not life history, predicts interpopulation differences in territorial aggression in a passerine bird. Anim. Behav.

[b88] Zanette LY, White AF, Allen MC, Clinchy M (2011). Perceived predation risk reduces the number of offspring songbirds produce per year. Science.

[b89] Zimmerman JL (1984). Nest predation and its relationship to habitat and nest density in dickcissels. Condor.

[b90] Zuur AF, Saveliev AA, Ieno EN (2012). Zero inflated models and generalized linear mixed models with R.

