# Supplementary material for: Partitioning the sources of demographic variation reveals density-dependent nest predation in an island bird population
Source: Ecol Evol. 2014 Jun 6;4(13):2738–48. doi: 10.1002/ece3.1127 (PMC4113296; doi:10.1002/ece3.1127)
Supplement: Supplementary file 1 [file ece30004-2738-SD1.docx]

Online Supplemental Information for:

Partitioning the sources of demographic variation reveals density-dependent nest predation in an island bird population

H. R. Sofaer, T. S. Sillett, K. M. Langin, S. A. Morrison, and C. K. Ghalambor

Table S1: Summary statistics describing mean annual variation in fecundity (total offspring fledged per pair). Data include all known nest attempts of each pair.

| Year | # of pairs | Proportion of pairs that failed (n) | Fecundity of successful pairs (± 1 SD) | Breeding density (territories/ha) | Precipitation (cm) |
| --- | --- | --- | --- | --- | --- |
| 2003 | 33 | 0.33 (11) | 2.4 ± 0.7 | 4.9 | 39.2 |
| 2004 | 30 | 0.43 (13) | 2.6 ± 0.8 | 4.8 | 18.5 |
| 2005 | 31 | 0.25 (8) | 3.2 ± 1.1 | 5.6 | 62.1 |
| 2006 | 25 | 0.72 (18) | 3.4 ± 0.8 | 4.9 | 20.6 |
| 2007 | 18 | 1 (18) | N/A | 5.6 | 9.1 |
| 2008 | 13 | 0 (0) | 3.8 ± 1.0 | 3.4 | 34.8 |
| 2009 | 31 | 0.87 (27) | 2.5 ± 1.0 | 6.3 | 19.4 |

Table S2: Our generalized mixed models showed strong support for density-dependent effects on daily nest survival; at higher breeding densities daily nest survival was lower. We considered an intercept-only model (.) and those with all additive combinations of five fixed effects: breeding density (bd), date, height (ht), precipitation (precip; equivalent to precip x-1 in Table 3) and nearest neighbor distance (nn). All models included a normally distributed random effect of year. Results are shown for the top ten models and the intercept only model.

| **Fixed-effect model** | **AIC_c_** | **ΔAIC_c_** | **weight** | **-2log(L)** | **k** |
| --- | --- | --- | --- | --- | --- |
| bd + date + ht | 548.02 | 0.00 | 0.24 | 537.99 | 5 |
| bd + date + ht + nn | 548.85 | 0.83 | 0.16 | 536.80 | 6 |
| bd + date + ht + precip | 549.15 | 1.14 | 0.13 | 537.11 | 6 |
| bd + date + nn | 549.42 | 1.40 | 0.12 | 539.39 | 5 |
| bd + date | 549.62 | 1.60 | 0.11 | 541.60 | 4 |
| bd + date + ht + precip + nn | 549.92 | 1.90 | 0.09 | 535.86 | 7 |
| bd + date + precip + nn | 550.44 | 2.43 | 0.07 | 538.40 | 6 |
| bd + date + precip | 550.75 | 2.73 | 0.06 | 540.71 | 5 |
| date + ht | 555.46 | 7.45 | 0.01 | 547.44 | 4 |
| date | 555.85 | 7.83 | 0 | 549.83 | 3 |
| . | 572.06 | 24.04 | 0 | 568.05 | 2 |

Table S3. Model selection results for the top ten Cormack-Jolly-Seber mark-recapture models. The model structures included an intercept-only model (.) and effects of: sex (s), breeding density (bd), wintering density (wd), precipitation during the winter prior to the March-March survival interval (precip x-1), and winter precipitation during the March-March survival interval (precip x). All models included sex and time effects on the resighting probability (*p*).

| **Model** | **AIC_c_** | **ΔAIC_c_** | **weight** | **-2log(L)** | **k** |
| --- | --- | --- | --- | --- | --- |
| φ(s + precip x-1) | 643.26 | 0.00 | 0.11 | 622.68 | 10 |
| φ(s) | 643.28 | 0.02 | 0.11 | 624.80 | 9 |
| φ(s + bd + precip x-1) | 643.74 | 0.48 | 0.09 | 621.04 | 11 |
| φ(s + bd) | 644.06 | 0.80 | 0.07 | 623.48 | 10 |
| φ(s + wd + precip x-1) | 644.26 | 1.00 | 0.07 | 621.56 | 11 |
| φ(s + precip x + precip x-1) | 644.39 | 1.13 | 0.06 | 621.68 | 11 |
| φ(s + bd + wd + precip x-1) | 645.00 | 1.74 | 0.05 | 620.17 | 12 |
| φ(.) | 645.23 | 1.97 | 0.04 | 628.85 | 8 |
| φ(s + precip x) | 645.34 | 2.08 | 0.04 | 624.76 | 10 |
| φ(s + wd) | 645.38 | 2.13 | 0.04 | 624.80 | 10 |

Figure S1: A bubble plot of the annual fecundity of warbler pairs in our study area showed no consistent pattern in the distribution of fecundity across space. An annual fecundity of 5-6 young was achieved via double brooding. Data are shown from all years except 2007, when a severe drought caused all monitored pairs to fledge no young.

Figure S2: A variogram of fecundity showing weak evidence of spatial autocorrelation within our study area. The number of pairs per spatial interval ranged from 69-563 pairs. Data are shown from all years except 2007, when a severe drought caused all monitored pairs to fledge no young.

Figure S3: Breeding density in a given year was not correlated with total November - April precipitation A) in the same year or B) in the previous year.

Figure S4: Breeding density was A) negatively correlated with breeding density in the previous year, and B) positively correlated with mean fecundity in the previous year.


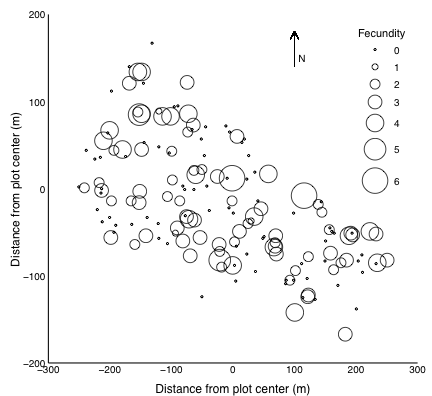


Figure S1


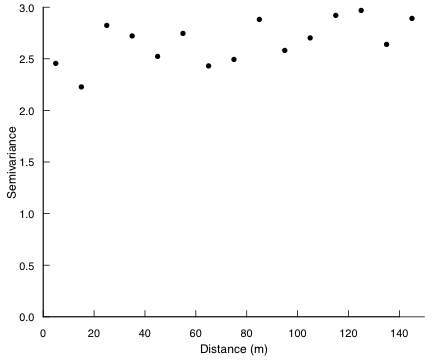


Figure S2


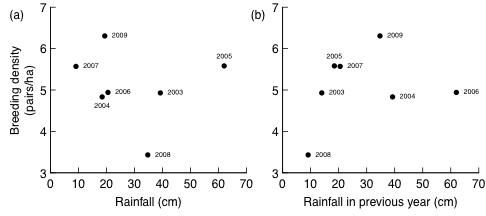


Figure S3


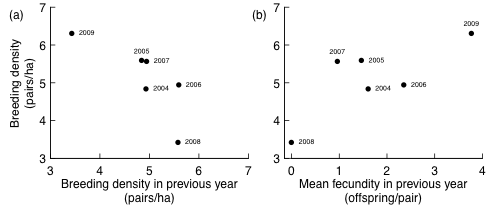


Figure S4
